# Peer Learning in Instrumental Practicing

**DOI:** 10.3389/fpsyg.2018.00339

**Published:** 2018-03-15

**Authors:** Siw G. Nielsen, Guro G. Johansen, Harald Jørgensen

**Affiliations:** Norwegian Academy of Music, Oslo, Norway

**Keywords:** peer learning, instrumental practicing, practicing and gender, practicing and genre, collaborative learning

## Abstract

In higher music education (HME), the notion of “private teaching, private learning” has a long tradition, where the learning part rests on the student's individual practicing between instrumental lessons. However, recent research suggests that collaborative learning among peers is beneficial in several aspects, such as sense of belonging, motivation and self-efficacy. This is consistent with the concept of vicarious learning. In this study, we conducted a survey among bachelor music students in church music, performance or music education programs enrolled in a music academy (*N* = 96), where parts of the questionnaire addressed peer learning and peer's influence on the students's instrumental practicing, and the degree of satisfaction with their practicing. These issues were seen in relation to gender, musical genre and study program. Overall, the students reported engaging in peer learning related to their instrumental practicing, to various degrees. This involved discussing practicing matters with peers, and practicing together with peers. However, student's reports of their views on peer learning, show that they perceive it more beneficial than the amount of time reported doing it would indicate. No significant gender differences were found, but students within improvised music/jazz engaged the most in peer learning, and church music students the least. Neither the degree of engaging in peer learning nor reported influence from peers correlated significantly with the degree of satisfaction. We discuss whether a general dissatisfaction is caused by being in a competitive learning environment combined with a privatized culture for learning. Finally, we suggest that collaborative forums for instrumental practicing within HME institutions can function as constructive and supportive arenas to enhance students learning and inner motivation.

## Background

Collaborative learning is steadily gaining more attention in educational research in music, and the concept of learning from peers is frequently addressed (Gaunt and Westerlund, 2013). Collaborative learning means that the subjects who take part in it, cultivate a shared understanding of goals and engage in joint problem-solving. As such, collaborative learning has a potential for increased reflection about canonized forms of interaction and hierarchies, according to Gaunt and Westerlund (2013, p. 4).

Peer learning, where students engage in mutual interactions with other students in order to learn, may have particular advantages as a context for collaborative learning. Hanken (2016, p. 365) argues for the strong benefits of peer learning in diverse disciplines, when she claims:

There is now a substantial body of research which indicates that learning from peers and together with peers can be beneficial in many ways for students in higher education studying diverse disciplines (…) It is, therefore, not surprising to see that many universities have implemented various strategies to enhance peer learning in practice.

According to social cognitive theory, learning from peers is important in constructing students' sense of personal efficacy in organizing and executing competent performance (Bandura, 1997, p. 3). Especially, this perspective emphasizes the impact of observational learning, where “seeing or visualizing people similar to oneself perform successfully typically raises beliefs in observers that they themselves possess the capabilities to master comparable activities” (p. 87). This is known as *vicarious learning* (Bandura, 1997). Thus, practicing with peers or discussing problem solving in practicing with peers may provide opportunities for music students to make such vicarious experiences, experiences that may prove beneficial to their own instrumental practicing.

Kokotsaki and Hallam (2007) found that higher education music students' perceived participating in collaborative music making to benefit their sense of belonging, making friendships, social skills, self-esteem, satisfaction, and thus facilitating development of personal identity and intrinsic motivation to mention some of the outcomes.

However, in music academies or conservatories the notion of “private teaching, private learning” has a long tradition, where the teaching part typically is performed in a “one-to-one teacher-student dyad” that takes place in the enclosed studio in work on the principal instrument (Davidson and Jordan, 2007, p. 730). The learning part rests on the individual practicing of the student between studio lessons, which might be seen as an expression of a privatized and individualized view on learning musical skills. For example, in a study of strategy use in instrumental practicing among first-year students in music academies, Nielsen (2004) found that the strategy “seeking help from peers” was used to a lesser degree than individual categories of strategies, such as cognitive and metacognitive strategies.

An individualized view on practicing also rests on the belief that “the teacher-student interaction in the lessons prepares for all the very private work that goes on by the learner in his or her practice” (Davidson and Jordan, 2007, p. 741). Although previous research in general reveals a strong relationship between the instrumental lesson and individual practice (Koopman et al., 2007), Koopman et al. (2007, p. 390) also conclude that minimal attention was paid to “strategies of executing and regulating practice” in lessons. This way the necessary self-reflection and regulation in the process of training did not develop in the students. Further, several studies indicate that although students may acquire important instrumental or vocal skills from the instrumental lesson, they do not necessarily learn how to transfer such skills to different contexts (Mills, 2002), nor to develop the independency needed for self-problem-solving (Kennel, 2002; Burwell, 2005).

This deficiency of the one-to-one learning context might be attributed to the hierarchical power relation between teacher and student (Nerland, 2004; Gaunt, 2010). In her study on students' perceptions of one-to-one tuition, Gaunt (2010) found that students seem passive in planning and evaluating their own development, waiting for their instrumental teachers' input. They often appropriate their teachers' views, which may preclude them from being critical and from developing their own artistic voice. Gaunt further suggests that if students actively seek inputs from other sources such as peers, this could represent a conflict in the student's relation to the teacher. Finally, Gaunt (2010) suggests that both students and teachers may need encouragement to consider the potential in peer learning.

However, it is important to keep in mind that it not necessarily the context itself that determines positive learning outcomes. For example, social relations among peer students may contain informal and hidden power hierarchies. If a student environment is experienced as hostile or competitive, then seeking advice or help from other students may be experienced as exposing one's weak sides, and thus uncomfortable or even threatening. Further, a mere observation of other students' active learning processes in for example master classes, does not in itself indicate that learning is collaborative if observers do not engage in a shared reflection. Likewise, it is not guaranteed that vicarious learning takes place if observing students for various reasons do not identify with the students they observe. Finally, although the power dynamics in the one-to-one learning context have been shown to have problematic aspects, such a teacher-student relationship can certainly enhance shared reflection and active learning in the student, depending on the pedagogical approach and attitudes in the teacher.

### Peer learning and genre

Some earlier studies address differences in practicing between musical genres (e.g., Creech et al., 2008; de Bézenac and Swindells, 2009; Sandgren, 2009). Creech et al studied—among other things—differences in individual practicing vs. peer learning between “classical” and “non-classical” (jazz, popular and folk music) students. These groups differed with regard to time spent on peer learning and individual practicing and how they ranked the importance of these learning activities. The classical students prioritized individual practicing, whereas jazz, pop, and folk music students reported learning in social settings. These settings included both practicing in ensembles, as well as listening to recordings and discussing learning together with peers. In line with this, Sandgren (2009) found that jazz students used more time to practice in ensembles than classical students. She also compared music students with psychology students on personality traits relevant to cooperation with others. Music students in general scored higher on openness toward influence from others and agreeableness than the other student group. This may be an important precondition for involvement in peer learning. In her study of how jazz students practice improvisation, Johansen (2016) found that to jazz students, collective practicing in bands was often considered a necessary learning arena for developing aspects of improvisational competence, such as musical responsiveness and collective agency (Edwards, 2009), intertwined with social dynamics in empathic creativity (Seddon, 2005). Such aspects may inform us about some reasons for genre related tendencies and differences in engagement in peer learning. However, processes of learning that involve peers may be of value for different purposes, including individual development, not only skills that are directly related to group performance.

### Peer learning and gender

In higher music education (HME), research has paid little attention to gender differences with regard to instrumental practicing, with some exceptions (Nielsen, 2004; Hallam et al., 2017). Hallam et al. (2017) found gender differences in use of practice strategies, concentration, and correction of errors during practice among pre-conservatoire students. On the other hand, Nielsen (2004) found no significant gender differences in first-year conservatoire students with regard to the use of learning strategies in individual practice sessions. However, female students were less self-efficacious than their male peers in instrumental practicing.

Zhukov (2012) investigated verbal and nonverbal interpersonal interactions between student and teacher in instrumental lessons, and found significant gender differences in nonverbal behavior. Males, whether teachers or students, used more deceiting visual cues to assert their dominance, while females, again both teachers and students, to a higher degree used courting visual cues to ingratiate themselves, whilst sending mixed signals. Her findings indicate that the dynamics in a social setting may differ according to the gender composition, which might influence the degree to which students engage in peer learning. Long (2013) studied students' experiences in instrument-specific master classes, and found that in this specific learning context, females experienced the master class as more intimidating and unfriendly than males. Hence, a social learning arena may not nurture students' positive development by default if this arena is experienced as competitive or negative.

Although efforts are being made to develop and understand collaborative learning practices in HME (Gaunt and Westerlund, 2013), there still is a need to explore and study collaborative learning in general, and peer learning in particular, in music academies, given the strong individualized conservatoire culture. Furthermore, whether students' loyalty toward their instrumental teacher represents a conflict regarding turning to peers as learning resources, also needs to be addressed.

## Research questions

The present study focuses on peer learning in instrumental practicing, and the research questions are:

1. To what degree do students report engaging in specific kinds of peer learning related to their instrumental practicing, and how is this related to gender, music genre and study program?
2. To what degree do students report specific kinds of peer learning as influential to their instrumental practicing, and how is this related to gender, music genre and study program?
3. To what degree does engaging in specific kinds of peer learning relate to how satisfied the students report being with their own practicing and their perceived success as performers?

## Methods

The survey study was conducted as an online study. As HME is a highly individualized activity involving busy students and few common meeting points, we assumed the use of a web based questionnaire—accessible to the students from their mobile phones or computers everywhere and at any time—as important in order to secure a satisfactory response rate.

### Participants

The participants were bachelor music students in church music, performance or music education programs enrolled in a music academy (*N* = 96). The sample included 53 women (53.5%) and 43 men (43.4%) (see Table 1 for a breakdown of the sample by study program and gender). By a breakdown by music genres 73 students (73.7%) were working in the Western classical music genre, 19 students (19.2%) in the improvised music/jazz genre, 6 students (6.1%) in the church music genre and 1 student (1%) in folk music (see Table 2 for a breakdown of the sample by music genre and gender). Because of tough competition for admission to these programmes, all students could be classified as advanced students. The students were recruited via e-mail and invited to respond to an electronic questionnaire. They were informed that their participation in the project was purely voluntary and that the study was approved by the *Data Protection Official of Norway*.

**Table 1:** A breakdown of the sample by study program and gender.

**Study program/Gender**	**Performance program**	**Music education program**	**Church music program**	**Total**
Female	33	16	4	53
Male	28	13	2	43
Do not want to provide	2	1	0	3
Total	63	30	6	99

**Table 2:** A breakdown of the sample by music genre and gender.

**Music genre/Gender**	**Improvised music/jazz**	**Western classical**	**Folk music**	**Church music**	**Total**
Female	6	42	1	4	53
Male	12	29	0	2	43
Do not want to provide	1	2	0	0	3
Total	19	73	1	6	99

### Questionnaire design

The questionnaire was structured into several themes, such as how the students perceived their practicing in general, time allotted to practicing, advice about practicing, planning of practicing, influence on their practicing, how they engage in peer learning in instrumental practicing, and how satisfied they were with their practicing and instrumental and vocal progress in general.

Students' reported *use of specific kinds of peer learning* were assessed with four items: (a) “I practice with my peers” where students rated themselves on a 4-point scale (1 = never; 2 = not so often as every week; 3 = every week; 4 = every day), (b) “Whenever practicing with your peers, how much time do you usually spend?” where students rated themselves on a 5-point scale (1 = < 1 h; 2 = 1–2 h, 3 = 3–4 h; 5 = 5–6 h; 5 = > 7 h) (c) “How often do you discuss practicing with your peers?” where students rated themselves on a 5-point scale (1 = never; 5 = always), and (d) “To what degree do you perceive discussing matters relating to your practicing with peers as beneficial?” where students rated themselves on a 5-point scale (1 = no degree; 5 = very large degree).

The students were asked to rate the *influences* on their practicing with five items: (a) “Your own influence on your instrumental practicing;” (b) “The influence of peers on your instrumental practicing;” (c) “The influence of your main instrumental teacher on your instrumental practicing;” (d) “The influence of other teachers on your instrumental practicing,” and (e) “The influence of books/magazines/web-pages to your instrumental practicing.” For each of the five items, students rated themselves on a 5-point scale (1 = none at all; 5 = very large).

The students were also asked to rate *how satisfied* they were with their own practicing and their own progress as performers (two items: “To what degree are you satisfied with your own practicing?;” “To what degree are you satisfied with your progress as a performer”) on a 5-point scale (1 = none at all; 5 = very large). See Table 3 for an overview of all variables.

**Table 3:** Means and standard deviations for the peer learning variables, the “influences on their practicing”—variables and the “satisfied with their own practicing and progress”—variables (*N* = 95).

**Variables**	**M**	***SD***
**PEER LEARNING VARIABLES**		
I practice with my peers	Ordinal	Ordinal
Time usually spend practicing with peers	Ordinal	Ordinal
How often do you discuss practicing with your peers?	3.20	0.83
To what degree do you perceive discussing matters relating to your practicing with peers as beneficial?	3.94	1.23
**INFLUENCES ON THEIR PRACTICING—VARIABLES**		
Your own influence on your instrumental practicing	4.33	0.69
The influence of peers on your instrumental practicing	3.04	0.89
The influence of		
-your main instrumental teacher	3.73	0.94
-other teachers	3.03	0.92
-books/magazines/web-pages	2.33	1.03
**SATISFIED WITH THEIR OWN PRACTICING AND PROGRESS—VARIABLES**		
Satisfied with your own practicing	3.60	1.11
Satisfied with your progress as a performer	4.23	1.24

### Procedure

The questionnaires were administered as a web based measure by a standard survey program package using the students' registered institutional email addresses. The students were informed that their participation in the project was purely voluntary, and only gender, study program/year and main instrument would be reported.

### Limitations

The participant group consisted of students from one (Norwegian) institution, and we acknowledge that cultural traits connected to this particular institution or geographical region may influence students' attitudes.

As the analyses are largely descriptive statistics, we clearly acknowledge that the correlations found do not indicate a causal relationship, and that these may be due to a whole host of other variables not represented in the questionnaire.

In the questionnaire, we defined “practicing together with peers” as extracurricular practice activity that students initiated themselves. However, we did not pose open questions as regards to what characterized such practice activities. Thus, the results reported regarding amount of time and perceived benefits may hide a potentially huge variety of activities. Not the least, it is possible that students from different genre cultures may have interpreted the questions differently. For example, Western classical students may differentiate between “practice” and “rehearsal”. Likewise, jazz students may engage in informal jamming activity which they don't necessarily define as practicing. Potentially, a great deal of collaborate discussions and reflections happen in activities which students may not have reported. It has previously been discussed in the literature that students' self-reports on practice behavior depends on what activities playing their instruments they count as “proper” practicing (Sloboda et al., 1996; Johansen, 2018). If students' shared talk about practicing happens in informal and relaxed settings, for example when having a break, it may be the case that they perceive it as less serious. Therefore, such verbal interactions may be underreported in this study, or reported but perceived less beneficial than students' more conscious and deliberate strategies to learn.

## Results

The first research question concerned to what degree the students did report engaging in specific kinds of peer learning related to their instrumental practicing. Peer learning can involve actually *practicing together*, and we asked the students to report how often they practiced with their peers. As can be seen in Table 4, a relatively large number of students (54.2%) did spend time practicing with peers every week, while 36.5% of the students did practice with their peers, but not so often as every week.

**Table 4:** Students reports on how often they practice with their peers (frequencies and percent in brackets) with a breakdown on gender, music genre and study program (*N* = 96).

	**Never**	**Not so often as every week**	**Every week**	**Every day**	**Total**
All	7(7.3)	35(36.5)	52(54.2)	2(2.1)	96(100)
Female	4(8)	20(40)	25(50)	1(2)	50(100)
Male	2(4.7)	14(32.6)	26(60.5)	1(2.3)	43(100)
Do not want to provide	1(33.3)	1(33.3)	1(33.3)	0	3(100)
Improvised music/jazz	0(0)	2(10.5)	15(78.9)	2(10.5)	19(100)
Western classical music	4(5.5)	32(44.4)	36(50)	0(0)	72(100)
Folk music	0(0)	1(100)	0(0)	0(0)	1(100)
Church music	3(75)	0(0)	1(25)	0(0)	4(100)
Performance program	4(6.5)	22(35.5)	34(54.8)	2(3.2)	62(100)
Music education program	0(0)	13(43.3)	17(56.7)	0(0)	30(100)
Church music program	3(75)	0(0)	1(25)	0(0)	4(100)

A chi square test was carried out in order to look into any differences in reported practicing with peers with regard to gender, music genre and study program. We found that music genre (*X*^2^ = 45.90; df = 9; *p* < 0.001) and study program (*X*^2^ = 31.02; df = 6; *p* < 0.001) were significantly related to how often the students practiced with their peers. 89% of the students in improvised music/jazz practiced with their peers at least once every week while only 50% of the students in Western classical music did the same. As can be expected, most students in church music did not practice with their peers at all (75%). There is no difference between students in music education and performance study programs regarding how often they practice with their peers. Further, we did not find any significant differences between female and male students with regard to how often they practiced with their peers. Hence, what music genre and study program the students belong to seemed to be the most important variable regarding this aspect of peer learning.

We also asked the students—whenever practicing with their peers—how much time they usually did spend. As can be seen in Table 5, most students practiced sessions of 1–2 h (with their peers).

**Table 5:** Students reports on time usually spent whenever practicing with peers (frequencies and percent in brackets) with a breakdown on gender, music genre and study program (*N* = 96).

	**Less than 1 h**	**1–2 h**	**3–4 h**	**Every day**	**Total**
All	14(14.6)	69(71.9)	12(12.5)	1(1.0)	96(100)
Female	7(14)	37(74)	5(10)	1(2)	50(100)
Male	5(11.6)	31(72.1)	7(16.3)	0(0)	43(100)
Do not want to provide	2(66.7)	1(33.3)	0(0)	0(0)	3(100)
Improvised music/jazz	1(5.3)	11(57.9)	6(31.6)	1(5.3)	19(100)
Western classical music	10(13.9)	57(79.2)	5(6.9)	0(0)	72(100)
Folk music	0(0)	0(0)	1(100)	0(0)	1(100)
Church music	3(75)	1(25)	0(0)	0(0)	4(100)
Performance program	9(14.5)	46(74.2)	6(9.7)	1(1.6)	62(100)
Music education program	2(6.7)	22(73.3)	6(20)	0(0)	30(100)
Church music program	3(75)	1(25)	0(0)	0(0)	4(100)

However, looking into any differences in reported length of sessions with regard to gender, music genre and study program, by carrying out a chi square test, we found that music genre (*X*^2^ = 32.29; df = 9; *p* < 0.001) and study program (*X*^2^ = 15.37; df = 6; *p* < 0.05) were significantly related to how long sessions the students practiced with their peers. Among the students in improvised music/jazz, 37% (of them) reported practicing longer sessions (3–4 or 5–6 h) while only 7% of the students in Western classical music reported the same lengths of sessions (when *practicing together*). This is as expected due to how the development of interactive communicative skills needs an interactive practicing context, for example jamming (improvising) together in bands.

Peer learning in instrumental practicing may also involve *discussing and reflecting* on practicing with peers, and thus, we asked the students to report how often they discussed practicing with their peers. As can be seen in Table 6, the general picture is that most students *do discuss matters* related to practicing with their peers as 37.9% of the students report that they *often* or *always* discuss practicing, and 43.2% report that they do this *sometimes*. These results indicate that students do use each other as resources for learning, but that some of them have an unexploited potential for involving more with their peers on this issue.

**Table 6:** Students reports on how often they discuss practicing with their peers (frequencies and percent in brackets) with a breakdown on gender, music genre and study program (*N* = 95).

	**Never**	**Seldom**	**Sometimes**	**Often**	**Always**	**Total**
All	2(2.1)	16(16.8)	41(43.2)	33(34.7)	3(3.2)	95(100)
Female	2(4.1)	8(16.3)	19(38.8)	18(36.7)	2(4.1)	49(100)
Male	0(0)	8(18.6)	20(46.5)	14(32.6)	1(2.3)	43(100)
Do not want to provide	0(0)	0(0)	2(66.7)	1(33.3)	0(0)	3(100)
Improvised music/jazz	0(0)	0(0)	10(52.6)	8(42.1)	1(5.3)	19(100)
Western classical music	1(1.4)	13(18.3)	31(43.7)	24(33.8)	2(2.8)	71(100)
Folk music	0(0)	0(0)	0(0)	1(100)	0(0)	1(100)
Church music	1(25)	3(75)	0(0)	0(0)	0(0)	4(100)
Performance program	0(0)	12(19.4)	27(43.5)	20(32.3)	3(4.8)	62(100)
Music education program	1(3.4)	1(3.4)	14(48.3)	13(44.8)	0(0)	29(100)
Church music program	1(25)	3(75)	0(0)	0(0)	0(0)	4(100)

In order to examine differences in reported *discussion of practicing* with peers with regard to gender, genre and study program, an analysis of variance (ANOVA) was conducted. Again, we found that music genre (*F* = 6.22; df = 3; *p* < 0.001) and study program (*F* = 7.42; df = 2; *p* < 0.001) were significantly related to these differences (how often the students had these kinds of discussions). Students in the improvised music/jazz genre reported having more discussion about practicing than the students in the Western classical music genre. All three study programs also differed significantly from each other (*p* < 0.001), and the students in the music education study program reported having more discussions with their peers than students in the other study programs. This might be due to these students' experiences of a more dialogue based education, in general, where sharing and reflecting on experiences on practice is an important part of the subject *Didaktik* in their music teacher education.

However, we were also interested in knowing to what degree the students perceived discussing matters relating to their practicing with peers as beneficial to them. As can be seen in Table 7, nearly half of the students (48.4%) reported that having such discussion were beneficial to them to a large or very large degree and 43.2% of the students reported that they were beneficial to some degree. These findings were found to be valid across gender, genres and study programs as we found no significant differences between these variables regarding this issue.

**Table 7:** Students reports on experienced benefits of discussing with peers (frequencies and percent in brackets) with a breakdown on gender, musical genre and study program (*N* = 95).

	**No degree**	**Little degree**	**Some degree**	**Large degree**	**Very large degree**	**Total**
All	1(1.1)	7(7.4)	41(43.2)	40(42.1)	6(6.3)	95(100)
Female	1(2.0)	4(8.2)	21(42.9)	19(38.8)	4(8.2)	49(100)
Male	0(0)	3(6.9)	18(41.9)	20(46.5)	2(4.7)	43(100)
Do not want to provide	0(0)	0(0)	2(66.6)	1(33.3)	0(0)	3(100)
Improvised music/jazz	0(0)	0(0)	11(57.9)	7(36.8)	1(5.3)	19(100)
Western classical music	0(0)	6(8.5)	29(40.8)	31(43.7)	5(7.0)	71(100)
Folk music	0(0)	0(0)	0(0)	1(100)	0(0)	1(100)
Church music	1(25)	1(25)	1(25)	1(25)	0(0)	4(100)
Performance program	0(0)	4(6.5)	24(38.7)	29(46.8)	5(8.1)	62(100)
Music education program	0(0)	2(6.9)	16(55.2)	10(34.5)	1(3.4)	29(100)
Church music program	1(25)	1(25)	1(25)	1(25)	0(0)	4(100)

We also found that there were a significant positive correlation between time spent discussing practicing with peers and perceiving these discussions as beneficial (*r* = 0. 35, *p* < 0.001), suggesting that students that spent more time discussing with peers also found this strategy to be more beneficial to them. Further, the difference between the means of these two variables in favor of perceiving the discussions as beneficial (*M* = 3.94, *SD* = 1.23) over spending time discussion practicing (*M* = 3.20, *SD* = 0.83), suggests that the general attitude of discussing practicing was perceived as being more beneficial than the amount of time reported doing it would indicate.

Our second research question concerned to what degree students did report specific kinds of peer learning (time spent practicing together and discussing practicing with peers) as being influential to their instrumental practicing. We asked the students to rate the influence of peers to their own instrumental practicing, and, as can be seen in Table 8, over half of the students (50.5%) reported that peers have had some influence on their practicing and 25.2% of the students reported that peers have had a large and very large influence. We found no significant differences on the rating of the influence of peers with regard to gender, genre or study program.

**Table 8:** Students reports on the influence of peers to their own practicing (frequencies and percent in brackets) with a breakdown on gender, musical genre and study program (*N* = 95).

	**No degree**	**Little degree**	**Some degree**	**Large degree**	**Very large degree**	**Total**
All	3(3.2)	20(21.1)	48(50.5)	18(18.9)	6(6.3)	95(100)
Female	2(4.1)	7(14.3)	23(46.9)	13(26.5)	4(8.2)	49(100)
Male	1(2.3)	12(27.9)	23(53.5)	5(11.6)	2(4.7)	43(100)
Do not want to provide	0(0)	1(33.3)	2(66.7)	0(0)	0(0)	3(100)
Improvised music/jazz	0(0)	5(26.3)	9(47.4)	3(15.8)	2(10.5)	19(100)
Western classical music	3(4.2)	12(16.9)	37(52.1)	15(21.1)	4(5.6)	71(100)
Folk music	0(0)	0(0)	1(100)	0(0)	0(0)	1(100)
Church music	0(0)	3(75)	1(25)	0(0)	0(0)	4(100)
Performance program	3(4.8)	14(22.6)	26(41.9)	14(22.6)	5(8.1)	62(100)
Music education program	0(0)	3(10.3)	21(72.4)	4(13.8)	1(3.4)	29(100)
Church music program	0(0)	3(75)	1(25)	0(0)	0(0)	4(100)

However, we were also interested in the relation between being influenced in their practicing from peers and the influence from their main instrumental teacher, and thus, we asked the students to rate the influence of their teacher. The influence from the teacher was rated as higher (*M* = 3.73, *SD* = 0.94) than the influence from peers (*M* = 3.04, *SD* = 0.89) (see Table 3), but we also found a significant positive correlation between these two variables (*r* = 0. 31, *p* < 0.01).

Our third research question concerned the relation between engaging in specific kinds of peer learning and the students' report on being satisfied with their own practicing and their perceived progress as performers. On an overall level, the students reported being more satisfied with their progress as performers (*M* = 4.23; *SD* = 1.24) than being satisfied with their own practicing (*M* = 3.60; *SD* = 1.11) (see Table 3).

However, we found that neither *spending time* practicing with peers nor *discussing practicing* with peers correlated positively to the degree the students were satisfied with their *own practicing* or their perceived *progress as a performer*. Likewise, neither reported perceived *influence from peers* nor their *perceived influence from teachers* correlated significantly to the degree the students were satisfied with their own practicing or to their perceived progress as a performer.

## Conclusion

Overall, the students reported engaging in peer learning related to their instrumental practicing, to various degrees. This involved sharing and discussing practicing matters with peers and spending time practicing with peers. Students within improvised music/jazz engaged the most in peer learning, and church music students the least. To various degrees they also reported their own practicing habits to be influenced by peers. Here, the music education students reported the highest peer influence, and we suggest this be due to these students' training in collaborative reflection. No significant gender differences were found in the amount of peer engagement, nor in students' experiences of peer or teacher influence, although we do not have data to inform us about gender differences in experienced social dynamics, as previous research suggests to be existing.

Based on the students' reports, we also found the general attitude that discussing practicing was perceived as more beneficial than the amount of time reported doing it would indicate.

An important finding is that there was an overlap in being influenced by both peers and the main instrument teacher. This finding is in contrast to the previously mentioned concern that a strong influence from the teacher might prevent students from seeking knowledge from other sources (Gaunt, 2010). Instead, the overlap might indicate an openness toward learning from others in general. Furthermore, if loyalty toward their teacher is not hindering a student from seeking knowledge from peers, it indicates that students perceive their relations to the teacher as trustful and non-authoritarian. If so, this might be a culture-specific feature.

Neither the degree of engagement in peer learning, nor the degree of influence from peers or teachers seemed to affect the degree to which students were satisfied with their own practicing. Careful conclusions should be drawn about this result, but several interpretations are possible. Firstly, receiving advice or support from teachers or reflecting on learning with peers can all contribute positively to a heightened awareness about practicing, but this does not necessarily lead to actual improved practice habits, if the students only know what to do, without putting it into action. If this is the case, then we need to know more about what factors can mediate changed habits. A second explanation might be that high achieving students in a competitive environment, where they perhaps are constantly reminded of ways to improve, attribute potential improvement to factors such as amount or quality of practicing. In that case, it is possible that many students have internalized a general sense of never doing enough work, regardless of the different learning strategies they do undertake and of their actual level. If this is the case, then a generalized sense of dissatisfaction with one's practicing efforts might be damaging for students' self-efficacy and thus motivation. Hence, we need to know more about how students can balance striving for improvement with appreciation of one's efforts and aptitudes at any stage of development. Creech et al. (2008) found that the jazz, pop and folk music students practiced mostly for the sake of joy, and were also the ones using peers as learning resources to the highest degree, which was confirmed in our study. The concept of vicarious learning (Bandura, 1997) refers to processes where observing others and imagining oneself in the observed situation holds a potential for enhanced learning. We propose that designing peer learning forums to enhance instrumental development in Western HME, may be a fruitful supplement to one-to-one tuition. In line with the theory of vicarious learning, it is fair to assume that identification with each other's learning processes through sharing experiences and reflections, can contribute to inner motivation and positive attitudes toward practicing, and thus stimulate a higher quality in students' practicing as well as enhance their transfer of learning to different contexts. However, as we have pointed out, not all collective learning forums may necessarily be experienced as constructive for all students, such as master- classes with a hostile or competitive atmosphere. We wish to suggest that peer learning forums, whether informal or formally instituted by institution, can function as a potentially constructive and supportive arena for reducing negative dimensions of competition and strengthen students' inner motivation in practicing. But for such benefits to take place, there is a need to know more about what characterizes specific interactive learning contexts that students find beneficial to participate in.

## Future research

The findings and the limitations of this study calls for further research on peer learning in instrumental development among music students. As mentioned in the method section, the sample of students in the study was limited, and specific institutional or geographical cultural traits experienced by these students may not be transferable to students in other regions. In concluding on the study's results, we implied that having an anti-authoritarian relationship between students and instrumental teacher may be such a cultural value. Future comparative studies between countries and between institutions may shed light on this issue.

Previously, we mentioned that the questionnaire may not have captured certain collaborative activities depending on how students perceived the term “practicing.” The nature and variation of such activities were not addressed, nor how and why (or why not) these are experienced as beneficial in students' development. For example, jazz students jamming together, classical string players practicing chamber music repertoire, or two students doing rhythmical exercises together may have different purposes for each group.

We suggest further studies to look more closely into the potential variety of peer learning activities, as well as the purposes students provide for such activities. With a more detailed and nuanced understanding of such activities, we will be able to more clearly crystalize the collaborative strategies students use or may benefit from using in their development. For educators in HME, this knowledge will be important to inform how we can design peer learning forums, and how we can enhance students' motivation, regardless of genre, to practice for the sake of joy.

## Ethics statement

This study was approved and carried out in accordance with the recommendations of the NSD - the Norwegian Social Science Data Services with written information to the sample and consent from all subjects.

## Author contributions

All three authors have designed and conducted the survey. In addition, SN and GJ have conducted the analysis of the data and written the article.

### Conflict of interest statement

The authors declare that the research was conducted in the absence of any commercial or financial relationships that could be construed as a potential conflict of interest.
